# Use of prescribed psychotropic drugs among medical students and associated factors: a cross-sectional study

**DOI:** 10.1590/1516-3180.2021.0566.R2.05012022

**Published:** 2022-08-12

**Authors:** Nicoli Abrão Fasanella, Clarissa Garcia Custódio, Júlia Santos do Cabo, Gabriel Sousa Andrade, Fernando Antônio de Almeida, Maria Valéria Pavan

**Affiliations:** I MD. Professor, Department of Public Health, Faculdade de Ciências Médicas e da Saúde (FCMS), Pontifícia Universidade Católica de São Paulo (PUC-SP), Sorocaba (SP), Brazil.; II Undergraduate Medicine Student, Faculdade de Ciências Médicas e da Saúde (FCMS), Pontifícia Universidade Católica de São Paulo (PUC-SP), Sorocaba (SP), Brazil.; III Undergraduate Medicine Student, Faculdade de Ciências Médicas e da Saúde (FCMS), Pontifícia Universidade Católica de São Paulo (PUC-SP), Sorocaba (SP), Brazil.; IV BSc, LPsy. Psychologist, Incorporee Instituto de Saúde, Guarapuava (PR), Brazil.; V MD, PhD. Full Professor, Department of Clinics, Faculdade de Ciências Médicas e da Saúde (FCMS), Pontifícia Universidade Católica de São Paulo (PUC-SP), Sorocaba (SP), Brazil.; VI MD, MSc, PhD. Professor, Department of Public Health, Pontifícia Universidade Católica de São Paulo (PUC-SP), Sorocaba (SP), Brazil.

**Keywords:** Mental health, Students, medical, Psychotropic drugs, Anxiety, Depression, Academic routine, Psychiatric diagnosis, Sleep and mental health

## Abstract

**BACKGROUND:**

The worldwide prevalences of anxiety and depressive disorders are 3.6% and 4.4%, respectively. Among medical students, many studies have indicated that the prevalences of these mental disorders vary between 19.7% and 47.1%, but there is a lack of information on psychotropic drug usage in this group of students.

**OBJECTIVE:**

To evaluate the prevalence of psychotropic drug use, adherence to therapy and main clinical and diagnostic indications relating to psychotropic drug use among medical students.

**DESIGN AND SETTING:**

Cross-sectional study at a Brazilian private university in the city of Sorocaba, state of São Paulo.

**METHODS:**

Observational analytical cross-sectional study, conducted during the second semester of 2019, through a semi-structured online questionnaire, answered by first to sixth-year medical students.

**RESULTS:**

Among the 263 participants (41.7% of the 630 enrolled students), the current prevalence of psychotropic drug usage was 30.4%. This prevalence increased over the course and 90.7% of the drugs were prescribed at regular medical consultations (85.5% by psychiatrists). The main indications for psychotropic drug usage were anxiety (30.0%), depression (22.8%), insomnia (7.2%), panic (5.3%) and attention deficit hyperactivity disorder (3.8%). Women were more likely to present diagnoses of depression and panic. Most of the participants used antidepressants and had good adherence to medications. Adequate sleep and regular physical activity were identified as protective factors against mental disorders.

**CONCLUSION:**

The prevalence of mental disorders among medical students is high, which justifies the use of psychotropic drugs. This study provides valuable information and recommendations for institutional educational actions to improve students’ mental health.

## INTRODUCTION

An individual’s mental health relates to biological, psychological, cultural and social factors, which contribute to the person’s adaptability, self-management and emotional wellbeing.^1^ Young adults experience rapid mental development, in which the social context is also deemed of great importance.^2^ In this regard, the mental health of university students has received greater attention in recent years, especially when concerning medical students.^3–5^

Due to the very nature of medical students’ education, greater requirements are placed on them regarding commitment, responsibility and academic performance.^3^ They usually have long study hours, with exposure to competitive environments and often sleep deprivation, which is far from the ideal learning environment.^4^ These factors may lead towards high levels of stress, which negatively influence both their physical and mental health.^3,4^

According to World Health Organization (WHO) data, the worldwide prevalence of depression in the population between 20 and 29 years old reaches 6% in women and 4% in men.^6^ Anxiety is the second most common mental disorder, with prevalence in the same age group of around 5% and 3% in women and men, respectively.^6^ In Brazil, 5.8% of the population are estimated to be affected by depression and 9.3% have anxiety.^6^

Among medical students worldwide, the prevalence of anxiety was estimated to be 33.8% in a meta-analysis.^3^ In Brazil, symptoms of depression, anxiety and stress can be found in 30% to 47% of medical students.^7,8^ Such symptoms can harm these students’ lives as well as their future patients. They may be debilitating and lead to worsening of these students’ academic and social performance.^5,9,10^

Use of psychotropic drugs can be a protective factor when well indicated, or may be another risk factor for mental health when misused. Studies carried out in recent years have revealed that the global prevalence of psychotropic drug use among young adults can vary from 6.5% to 22.3%.^11–14^ In Brazil, the prevalence of psychotropic drug consumption among medical students is similar and varies from 10.7% to 22.6%, depending on the class of such psychotropic drugs that is evaluated.^15,16^

## OBJECTIVE

The objective of this study was to evaluate the prevalence of psychotropic drug use, the degree of therapeutic adherence and the main clinical indications relating to use of psychotropic drugs among students in the first to the sixth year of the medical course at a Brazilian private university.

## METHODS

### Study design

This was a cross-sectional analytical observational study, carried out during the second semester of 2019 at the Faculty of Medical and Health Sciences of the Pontifical Catholic University of São Paulo, located in the city of Sorocaba.

The students received a semi-structured, self-applicable questionnaire, built by the authors, which was sent out and answered electronically. The predefined questions of the questionnaire were organized in two parts. The first part of the questionnaire consisted of asking for personal and social data, with questions about age, gender, year of the course, whether the student had a scholarship or study funding, with whom the student was living and the student’s city of origin. The second part of the questionnaire sought to identify whether the students were using (or had ever used) psychotropic drugs and asked about the determinants for use of such medication. To assess treatment adherence, the Measuring Instrument of Treatment Adherence (MITA) was used, as adapted and validated for Brazilian Portuguese by Borba et al.^17^ The criterion for considering that the individual’s adherence to treatment was adequate is a score of 5 or 6 (on a scale of 1 to 6).^17^

The questionnaire was built and sent through Google Forms. The link to access the informed consent statement, as well as the questionnaire, was sent by email. Access to the questionnaire necessarily depended on the acceptance of the consent statement, which was considered to represent an electronic signature.

### Study participants

All medical students aged 18 and over, in any year from the first to the sixth, were invited to participate in the study. Out of the 630 medical students enrolled in 2019, 268 responses were obtained.

The sample size was calculated with tolerance of an error of 5%, a confidence level of 95% and great heterogeneity of the population (50%).^18^ According to these criteria, considering the total number of students enrolled in the medical course in the second semester of 2019, it was found that a representative sample should have at least 244 participants.

### Statistical analysis

The data were analyzed using the SPSS software (PASW Statistics for Windows: version 18.0; Chicago, Illinois, United States). Initially, a descriptive statistical analysis of the social characteristics of the studied population was performed. To explore the interrelation between the variables, nonparametric statistical tests were used, adopting the confidence level of 95%. These tests were performed to assess whether to reject the null hypothesis (that the variables were independent). Simple binary logistic regression models were elaborated to assess the impact of the progression within the medical course on the dependent variables of psychotropic drug use and psychiatric diagnosis, which were both binary variables. In this study, use of psychotropic drugs was the main variable of interest, while the covariables or independent variables consisted of adherence to treatment, year of progression in the medical course, sleeping hours and sociodemographic characteristics.

The statistical analysis aimed to describe the frequency of psychotropic drug use among the participants and, additionally, relate this to psychiatric diagnoses and treatment adherence. Thus, chi-square tests were conducted to identify whether there were any associations between pairs of categorical variables, and the phi (ϕ) statistic was used as a measurement of effect size. Fisher’s exact test was used to investigate the significance of associations between pairs of categorical variables when the sample size was small (which would break the premises of chi-square). The Mann-Whitney U test was used to compare pairs of groups of values without assuming that these values were normally distributed, using Cliff’s delta (d_Cliff_) as a measurement of effect size. Spearman’s correlation test was used to measure the strength of association between pairs of variables.

### Ethical considerations

This research project and the informed consent statement that would be used were submitted to the Research Ethics Committee of the Faculty of Medical and Health Sciences of PUC-SP and the study only started after approval had been granted. Date of approval: May 14, 2019. Certificate of Presentation for Ethical Appreciation (CAAE): 11130019.8.0000.5373.

## RESULTS

### Participating students’ profile

The final sample was composed of 263 participants out of a universe of 630 students (41.7%). The number of participants in each course year did not differ among the participants (chi-square test, χ^2^; P = 0.833) The proportion of women in the sample was significantly higher than that of men (χ^2^; P < 0.001), and was also statistically higher than the proportion of female physicians aged up to 29 years in practice in Brazil (χ^2^; P = 0.002).^19^ The participants’ profile is described in Table 1.

**Table 1.** Participants’ profile

Parameter evaluated	Results observed (n = 263)
Age (mean ± SD)	22.9 ± 2.7 years
Gender	66.5% female
Sexual orientation	83.7% heterosexual 7.6% homosexual 7.2 % bisexual 1.2% other
Hometown	91.3% from other municipalities
Funding/scholarship status	35% had scholarship
Healthcare insurance	75.7%
Regular physical activity^1^	65.8%
Daily sleeping hours	0.4%, less than 4 hours 30.0%, 4 to 6 hours 63.5%, 6 to 8 hours 6.1%, over 8 hours

^1^ At least three times/week; SD = standard deviation.

Thirty-five percent of the participants had some type of scholarship. Students originally from the city of Sorocaba were more likely to have received funding (Sorocaba 69.6%; other municipalities 31.7%; χ^2^, P < 0.001).

Participants who practiced regular physical activity reported having a greater number of hours of sleep (Mann-Whitney U test; P = 0.008; with a small effect size, d_Cliff_= 0.167).

### Use of psychotropic drugs among the participants

Out of the 263 students included in the study, 109 (41.4%) reported having used psychotropic drugs at some time in their lives and 80 students (30.4%) reported that they were currently using at least one psychotropic drug. The characteristics of the psychotropic drug use among the participants are described in Table 2.

**Table 2.** Characteristics of psychotropic drug use among participants

Participants (n = 263)	Number (%, 95% CI)
**Use of psychotropic drugs at some time in life**	109 (41.4%, 35.2-47.6%)
**Use of psychotropic drugs currently**	80 (30.4%, 25.1-35.7%)
1 psychotropic drug	65 (81.8%, 72.8-90.3%)
2 psychotropic drugs	8 (10.4%, 3.9-18.2%)
3 psychotropic drugs	6 (7.8%, 2.3-14.3%)
**Classes of psychotropic drugs used:** **Antidepressants** Selective serotonin reuptake inhibitors Serotonin and norepinephrine reuptake inhibitors Norepinephrine and dopamine reuptake inhibitors Tricyclic antidepressants **Benzodiazepine anxiolytics** **Mood stabilizers** **Amphetamine-derived central nervous system stimulants**	50 (19.0%, 12.2-21.5%) 13 (4.9%, 2.3-7.3%) 8 (3.0%, 0.8-4.9%) 4 (1.5%, 0.0-2.7%) 13 (4.9%, 1.8-6.2%) 10 (3.8%, 1.6-6.3%) 7 (2.7%, 1.1-4.6%)
**Most common psychotropic drugs used:** Escitalopram Fluoxetine Bupropion Desvenlafaxine Sertraline Clonazepam	21 (8.0%, 4.9-44.4%) 10 (3.8%, 1.9-6.4) 8 (3.0%, 1.1-5.3) 7 (2.7%, 0.8-4.6%) 7 (2.7%, 0.8-4.9%) 7 (2.7%, 0.8-4.8%)
**Medical prescription at regular follow-up** Psychiatrist Other specialties General practitioner	99 (90.7%, 84.4-95.4%) 93 (85.5%, 77.6-91.9%) 13 (11.8%, 6.4-19.4%) (2.7%, 0.0-5.2%)

CI = confidence interval.

The great majority of the participants reported that psychotropic drugs were first prescribed by the doctors with whom they regularly had consultations (90.7%). Among these, 85.5% reported having been followed up by psychiatrists, 12.4% by doctors of other specialties and 2.1% by general practitioners. As the medical students progressed along the medical course, they were more likely to be followed up by psychiatrists than by any other specialist (Mann-Whitney U test; P = 0.01; d_Cliff_= 0.452). Among the 9.3% of the students who had received prescriptions of psychotropic drugs from a professional other than the one who was their follow-up doctor, 30% reported receiving a prescription from their parents, 30% from a friend and 40% from “others”.

Among the students using psychotropic drugs, 47.5% had already received medication prescriptions from doctors other than those who were following them up (40.4% from family members, 21.3% from teachers, 22.3% from friends and 16% from residents). Receiving prescription drugs from other professionals is a factor associated with lack of healthcare insurance (χ^2^, P = 0.003; ϕ = 0.293).

As reported by the participants, the clinical indications for psychotropic drug use are shown in Table 3.

**Table 3.** Main clinical indications for psychotropic drug use among medical students

Clinical indications	Percentage (95% CI)
Anxiety	30.0% (24.7%-36.0%)
Depression	22.8% (17.8%-28.3%)
Insomnia	7.2% (4.2%-10.6%)
Panic	5.3% (2.7%-8.4%)
Attention deficit hyperactivity disorder	3.8% (1.5%-6.4%)
Bipolar affective disorder	1.5% (0.4%-3.1%)
Compulsion	1.5% (0.4%-3.1%)
Obsessive-compulsive disorder	1.1% (0.0%-2.6%)
Weight loss	1.1% (0.0%-2.7%)
Other	3.4% (1.5%-5.7%)

CI = confidence interval.

**Graph 1:**
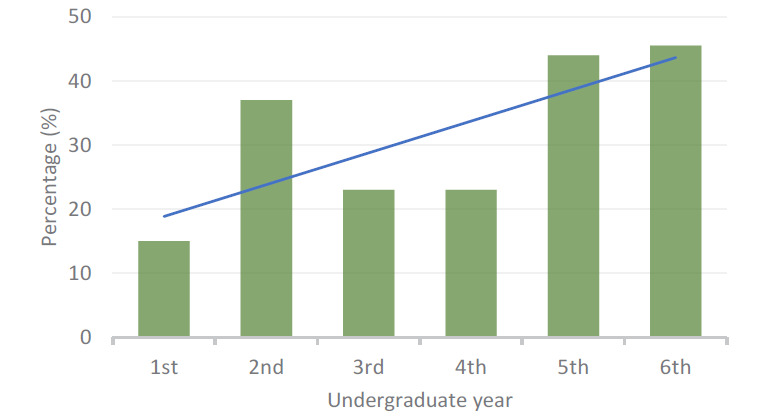
Prevalence of psychotropic drug use among medical students.

Among the participants who were currently using psychotropic drugs, 31.3% started doing this before entering the medical course, 10.0% started in the first year of medical school, 13.8% in the second, 16.3% in the third, 13.8% in the fourth, 12.5% in the fifth and 2.5% in the sixth. As shown in Graph 1, the prevalence of psychotropic drug use reached 45% of these medical students in the last two years of their course.

To assess the impact of the progression within the medical course on the odds of psychotropic drug use, simple binary logistic regression models were constructed. As depicted in Table 4, progression within the medical course predicted psychotropic drug use. According to the model, it was estimated that the chance that medical students would use psychotropic drugs increased by 27.1% (95% confidence interval, CI: 8.7-48.5%; P = 0.003) for each year of progression along the course. The same model also showed that the students’ age also predicted psychotropic drug use. As seen in Table 4, the odds of using psychotropic drugs increased by 14.8% each year (P = 0.011).

**Table 4.** Simple binary logistic regression model to assess the impact of progression along the medical course on the dependent variables (psychotropic drug use and psychiatric diagnosis)

Dependent variable	Predictor	β	Seβ	Wald’s χ^2^	df	P-value	Exp(β)	Exp(β) (Lower 95% CI)
**Psychotropic drug use**	**Course year**	0.239	0.8	9.059	1	0.003**	1.271	1.087
	Intercept	-1.67	0.321	27.022	1	< 0.001	0.188	
	**Age**	0.138	0.54	6.476	1	0.011*	1.148	1.032
	Intercept	-3.896	1.268	9.433	1	0.002	0.02	
**Psychiatric diagnosis**	**Course year**	0.236	0.096	5.964	1	0.015*	1.266	1.048
	Intercept	-1.958	0.387	25.612	1	< 0.001	0.141	
	**Age**	0.99	0.61	2.643	1	0.104	0.105	0.98
	Intercept	-3.293	1.42	5.381	1	0.02	0.037	

β coefficient; Seβ = coefficient standard error; CI = confidence interval; Wald’s χ^2^ = test statistics for the variables in the equation; df = degrees of freedom; Exp(β) = the average proportion at which the odds of using a psychotropic drug or having a psychiatric diagnosis increases for each unit increase in course year or age. *Significant, within 95% confidence interval; **significant, within 99% confidence interval.

In comparing the prevalence of psychotropic drug use among third and fourth-year students with that of fifth and sixth-year students, a statistically significant difference was observed, such that it was higher in later years (χ^2^, P = 0.003). Students in the last two years of the medical course, compared with those in the first two years, were more likely to use mood stabilizers (Fisher’s exact test, P = 0,034), escitalopram (P = 0.037), clonazepam (P = 0.026), alprazolam (P = 0.016) and other psychotropic drugs (P = 0.012). It was also observed that anxiety (χ^2^, P = 0.029) and panic (χ^2^, P = 0.009) were reported as the main clinical indications for use of psychotropic drugs among medical students in the fifth and sixth years, with no other significant relationships.

### Treatment adherence

Regarding the responses in the questionnaire relating to adherence to medication treatment, it was found that 64.5% (95% CI: 46.9-80.6%) of the participants who had used psychotropic drugs had adequate adherence to the treatment, while 35.5% (95% CI: 19.4-53.1%) were poorly adherent. The participants’ mean score was 5.16 (95% CI: 4.9-5.4).

An association was observed between the symptom “discouragement” and non-adherence to treatment (Fisher’s exact test, P = 0.031). A negative correlation was found between the sleep hours scale and carelessness with medication schedules, such that those who slept less tended to forget to take the medication more often (Spearman’s correlation test, r = 0.353; P = 0.03). No correlations were found for the course year or participant age in relation to carelessness with psychotropic drug use or to general treatment adherence.

### Psychiatric disorders as indicative for use of psychotropic drugs

Altogether, 25.5% of the participants reported having some kind of psychiatric diagnosis (95% CI: 18.4-30.4%). Among these diagnoses, the following were mentioned: anxiety disorders (11.4%), depressive disorders (11.0%), attention deficit hyperactivity disorder (1.9%), obsessive compulsive disorder or compulsion (0.8%) and personality disorders (0.4%).

Like what was observed regarding psychotropic drug use, participants who had progressed further within the medical course were more likely to report having a psychiatric diagnosis (Mann-Whitney U test, P = 0.012). As shown in Table 4, according to the binary logistic regression model, the chance of reporting a psychiatric diagnosis grew by approximately 26.6% (95% CI: 4.8 - 52.9%; P = 0.015) for every additional year of the students’ progression along the course, such that this chance reached 42% among participants in the fifth year.

Considering the prevalence of specific disorders over the years of the course, the only significant association found was depressive disorder, which tended to increase as the course progressed (Mann-Whitney U test, P = 0.041; d_Cliff_= 0.229;)

It was also observed that participants with psychiatric diagnoses made by a doctor reported having fewer hours of sleep than participants who did not report any psychiatric diagnosis (Mann-Whitney U test, P = 0.024; d_Cliff_= 0.179). Among the participants who reported sleeping for less than six hours a night, 56.4% also reported having a psychiatric diagnosis, but no association was found with any specific disorder.

It was found that women were more likely to be diagnosed with depressive disorder (χ^2^, P = 0.021; ϕ = 0.144) and to have symptoms like distress (χ^2^, P = 0.046; ϕ = 0.123), discouragement (χ^2^, P = 0.005; ϕ = 0.174) and irritability (χ^2^, P = 0.001; ϕ = 0.213). Women were also more likely to report panic as a motivator for use of psychotropic drugs (χ^2^, P = 0.04; ϕ = 0.131).

Students with depressive disorders were more likely to report feeling sadness (χ^2^, P < 0.001; ϕ = 0.328), discouragement (χ^2^, P < 0.001; ϕ = 0.238), insomnia (χ^2^, P = 0.004; ϕ = 0.189), a desire to remain left alone (χ^2^, P < 0.001; ϕ = 0.291), persecuted or watched (χ^2^, P < 0.001; ϕ = 0.268) or irritability (χ^2^, P = 0.003; ϕ = 0.188). They were also less likely to engage in regular physical activity (χ^2^, P = 0.014; ϕ = -0.155).

### Use of illicit drugs

Overall, 28.9% (95% CI: 24.0-34.8%) of the participants reported using some illicit drug. Marijuana use was reported by 25.5% (95% CI: 20.5-31.3%), methylenedioxymethamphetamine (MDMA) by 10.3% (95% CI: 6.6-14.6%), hallucinogens by 3.0% (95% CI: 1.1-5.3%), inhaled drugs by 3.0% (95% CI: 1.1-5.3%) and amphetamines by 1.1% (95% CI: 0.0-2.6%). Participants who reported using inhaled drugs slept less than those who did not report using them (Mann-Whitney U test, P = 0.004; d_Cliff_= 0.512). Use of illicit drugs was not associated with use of psychotropic drugs, or with the presence of any known mental disorder among the participants.

## DISCUSSION

The prevalence of mental disorders found in the sample was similar to that found in the literature.^8,10,14,20^ Regarding the use of psychotropic drugs, the prevalence found was as follows: 41.4% of the medical students had used psychiatric drugs at some time in their lives and 30.4% were using it at the time of the study. This prevalence was approximately five times higher than in the general population, even when compared with young students in pre-university courses, and was usually related to a higher risk of suffering from stress.^11–13,21^

The high percentage of medical students who use or have used psychotropic drugs at some time may indicate greater psychological distress among these individuals, considering that they are exposed to stress factors that can contribute to worsening of mental disorders. Vasconcelos et al. also observed a high prevalence of anxiety and depression among medical students in the city of Recife.^9^ In their study, anxiety was associated with use of psychotropic drugs, and depression with use of illicit drugs.^9^

As observed in our study sample, 69% of the participants who used some type of psychotropic drug started to do this at some point during the course. As the course progressed, the chance of receiving a psychotropic drug prescription increased by 27% annually, such that in the sixth year these prescriptions were most prevalent. It is in the last year of the course, which is also the last of the clinical years, that students experience the greatest university demands, with the greatest workload and responsibility in practical internship, and the closest contact with patients.^20^

Furthermore, in the first years of the course, there is great anxiety about the unknown: adaptation to entering university, moving to another city and adapting to new teaching methods, great changes in family dynamics, new colleagues and new teachers.^7^ As reported by Vasconcelos et al.,^9^ being from the region where one studies and living with one’s family are mental health protective factors. Among the students evaluated, more than 90% came from other municipalities and many of them were living on their own, which could partially explain the findings.

In the third and fourth years of the course, students are experiencing the best period of the course, at a time when they have become better adapted to and familiarized with their study needs and educational activities.^7^ On the other hand, in the fifth and sixth years, there is an increase in the prevalence of psychotropic drug use and psychiatric diagnoses. The last few years are characterized by intensification of practical activities, closer contact with patients and greater responsibility, as students approach the time of graduation and of assuming the life of a medical professional, and the great difficulty in accessing specializations, which are all possible stressors.^7,8,20^

Our study also identified that the profile of prescribers for these students, in 90% of the cases, was the doctor with whom the students undertook their regular healthcare. Psychiatrists were the main choice of specialist, thus indicating that these students at a private university had access to specialists for their mental healthcare and treatment. According to Quintana et al.,^12^ prescription of psychotropic drugs for the general population was done mainly by general practitioners, followed by psychiatrists. The data obtained in the present study can be interpreted as a specific characteristic of the sample presented here, which consisted of a population in which the majority had healthcare insurance. However, these data may also indicate that there is less stigmatization of the specialty of psychiatry among medical students.

Students who did not have healthcare insurance, who were a minority within the sample, were the ones who most received prescriptions from professionals other than those with whom they undertook their regular monitoring. In addition to being an ethical transgression by the prescriber, this practice may also be a risk factor, since students who are not under regular medical supervision, and receive prescriptions from someone other than their regular doctor, are not integrated into a means of broadened care in which an appropriate doctor-patient relationship is established, with therapeutic bonds and periodic reevaluations. Thus, these students are exposed to a situation of perpetuation of prescriptions that are sometimes inadequate.^22,23,24,25,26^

It is also necessary to consider that at least 35.5% of the study participants who were using some type of psychotropic drug did not have adequate adherence to treatment, which may have been a factor in worsening their mental health. Irregular use of these medications is associated with a greater risk of aggravation of the individual’s depressive symptoms or postponement of clinical improvement, thereby worsening the prognosis.^23,24^

In the present study, no significant difference in adherence to treatment was observed between students in different years of the course. This may be an indication that there is no relationship between technical knowledge and adherence to treatment. Instead, this may indicate that medical students have a profile that leads them to neglect their own health, or even to suffer from the stigma of mental illness or the need to use psychotropic drugs, which would therefore lead them not to use medications correctly.^27^

Antidepressants are the most prescribed psychotropic drugs in the general population and are the medications of choice for treatment of anxiety and/or depressive disorders.^12,28^ In the population studied, antidepressants were also the most prescribed drugs, representing about 25% of prescriptions. A significant portion (52.8%) of the students who used a psychotropic drug reported having anxious or depressive symptoms, and identified this as the main reason for using this class of medication. This was followed by insomnia, reported by 7.2%.

The students’ sleep pattern seemed adequate for more than 60% of the participants, who reported having more than six hours of sleep per night, which is one of the criteria for assessing sleep quality.^29^ Insomnia is a risk factor and, at the same time, a sign of mental health impairment.^30,31^ Among the participants in the present study, it was observed that having less sleep was a risk factor for a diagnosis of some psychiatric disorder and for incorrect medication use. In other words, students who said that they were sleeping for fewer hours a night had higher prevalence of mental disorders and tended to forget to take their psychotropic drugs.

Also, among the participants in this study, regular physical activity was seen to be a protective factor against depression diagnosed by a doctor. Regular physical activity is a known protective factor against depression, as already reported.^32^ Furthermore, in the present study, individuals who practiced regular physical activity were also sleeping for longer times.

The prevalence of use of illicit substances was close to what has been identified in the literature for this age group.^21,33,34^ The most-used substance was marijuana, which is a substance for which usage has been correlated with increased risk of suicide, in addition to other cognitive impairments.^35^

Our study had some limitations, considering that it was conducted in a single private medical school. Therefore, although the sample size did represent the whole population of medical students in the institution, with a 5% tolerance error and 95% confidence level, phenomena with low prevalence cannot be evaluated in these circumstances.

## CONCLUSION

This study showed the high prevalence of psychotropic drug use among medical students. The clinical indication for use of these drugs was psychological distress, and anxiety, depression, insomnia and panic were the most prevalent disorders. It also demonstrated that the prevalence of psychiatric disorders and the use of psychotropic drugs increase over the years of the course, and that there may be a reduction in the third and fourth years. The risk factors for mental disorders comprised reduced hours of sleep, lack of regular physical activity, progression along the medical course and female gender. Women were more likely to receive diagnoses of depressive disorder and panic, and to present symptoms such as distress, discouragement and irritability. Regular physical activity and a good sleep pattern were protective factor against mental disorders.
